# Chicoric Acid: Natural Occurrence, Chemical Synthesis, Biosynthesis, and Their Bioactive Effects

**DOI:** 10.3389/fchem.2022.888673

**Published:** 2022-06-23

**Authors:** Min Yang, Chao Wu, Tianxi Zhang, Lei Shi, Jian Li, Hongbao Liang, Xuzhen Lv, Fengtang Jing, Lu Qin, Tianlun Zhao, Chenxi Wang, Guangxu Liu, Shuai Feng, Feng Li

**Affiliations:** ^1^ Teaching and Research Office of Chinese Medicines authentication, College of Pharmacy, Shandong University of Traditional Chinese Medicine, Jinan, China; ^2^ Department of Pharmaceutical Preparation Technology, Department of Pharmaceutical Engineering, Shandong Drug and Food Vocational College, Weihai, China; ^3^ Grade Three Laboratory of Traditional Chinese Medicine Preparation, Department of Pharmacy, Affiliated Hospital of Shandong University of Traditional Chinese Medicine, Jinan, China; ^4^ Lunan Pharmaceutical Group Co., Ltd., State Key Laboratory of Generic Manufacture Technology of Chinese Traditional Medicine, Linyi, China

**Keywords:** chicoric acid, biosynthesis, chemical synthesis, natural occurrence, content detection, bioactive effects

## Abstract

Chicoric acid has been widely used in food, medicine, animal husbandry, and other commercial products because of its significant pharmacological activities. However, the shortage of chicoric acid limits its further development and utilization. Currently, *Echinacea purpurea* (L.) Moench serves as the primary natural resource of chicoric acid, while other sources of it are poorly known. Extracting chicoric acid from plants is the most common approach. Meanwhile, chicoric acid levels vary in different plants as well as in the same plant from different areas and different medicinal parts, and different extraction methods. We comprehensively reviewed the information regarding the sources of chicoric acid from plant extracts, its chemical synthesis, biosynthesis, and bioactive effects.

## 1 Introduction

**FIGURE 1:**
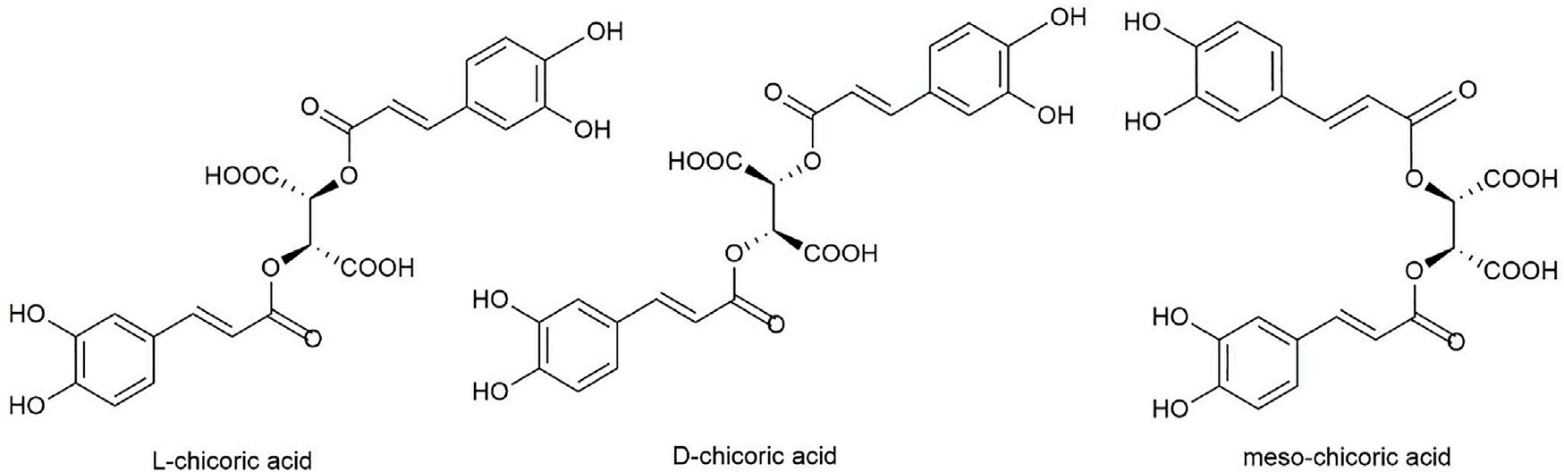
Three optical structures of chicoric acid.

Current research on chicoric acid focuses primarily on medicinal, chemical, natural agricultural production, and food and accounts for 50%, 18%, 13%, and 18%, respectively. Chicoric acid belongs to caffeic acid derivatives and its molecular formula is C_22_H_18_O_12_. Chicoric acid is soluble in ethanol, methanol, dioxane, acetone, and hot water; slightly soluble in ethyl acetate and ether; and insoluble in ligroin, benzene, and chloroform (Scarpati and Oriente, 1958). There are two chiral carbon atoms in this structure, so chicoric acid is divided into levorotatory-chicoric acid (L-chicoric acid), dextrorotatory-chicoric acid (D-chicoric acid), and meso-chicoric acid (meso-chicoric acid) (Figure 1).

**FIGURE 2:**
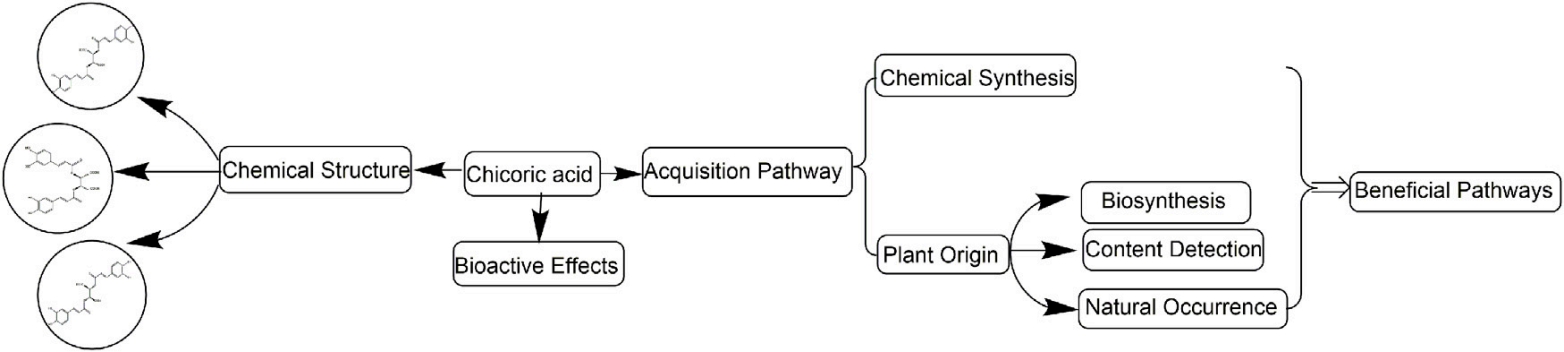
Chemical structure, bioactive effects, and acquisition pathways of chicoric acid.

Chicoric acid is a rare and valuable functional food ingredient with no obvious dose dependence, no overdose side effects, and no contraindications and drug interactions. Chicoric acid has been widely used in medicines, nutritional supplements, and health foods due to its promising pharmacological effects in regulating glucose and lipid metabolism; anti-inflammatory, antioxidant, and anti-aging properties, and against digestive system diseases (Peng et al., 2019). *Echinacea purpurea* (L.) Moench, the main plant material of chicoric acid, has a 400-year history of use in Europe and America. However, the shortage of chicoric acid limits its further development and utilization. So, this article focuses on systematically summarizing chicoric acid sources, such as chemical synthesis and biosynthesis, and further elaborates on resource distribution and content of chicoric acid in different plants, aiming to find a beneficial pathway of chicoric acid production for further development and utilization (Figure 2).

## 2 Natural Occurrence of Chicoric Acid

Plants containing chicoric acid are rich in resources and widely distributed, so chicoric acid has been utilized as an alternative medicine or a food supplement for quite some time (Street et al., 2013). Chicoric acid levels in different plants and different parts of the same plant often differ significantly. However, there are few reports on chicoric acid levels and different plant resource distributions. At present, *E. purpurea* is the main crude material for the extraction of chicoric acid, but the shortage of crude material limits further development and utilization. In order to solve that problem, it is necessary to analyze the resource distribution and chicoric acid levels in different plants.

### 2.1 Plants the Principal Sources of Chicoric Acid

At least 25 families, 63 genera and species, in the plant kingdom contain chicoric acid (Grignon-Dubois and Rezzonico, 2013), there is no substantial guidance due to the lack of detailed information on plant resources. For the convenience of discussion, this article has divided chicoric acid–containing plants into angiosperms, ferns, and other categories (Table 1) and discussed the resource distribution, historical changes, and usage of chicoric acid.

**TABLE 1:** Plants that are the principal sources of chicoric acid.

Plant names	Family	Genus	Resource distribution	Morphological classification	Plant parts	References
*Echinacea purpurea* (L.) Moench	Asteraceae	*Echinacea*	North America and China	Perennial herb	Aerial parts and roots	Shah et al. (2007); Wills and Stuart (1999); Wang et al. (2016)
*Pterocypsela laciniata* (Houtt.) Shih	Asteraceae	*Pterocypsela*	East Asia and Southeast Asia	Perennial herb	Leaves	He (2012); Ke et al. (2015)
*Cichorium intybus* L.	Asteraceae	*Cichorium*	Mediterranean region and Southwest Asia	Perennial herb	Aerial parts and roots	Carazzone et al. (2013); Mascherpa et al. (2012); Zhou (2014); Xu (2008)
*Lactuca sativa* L.	Asteraceae	*Lactuca*	Temperate areas	Annual or biennial plant	Lettuce head and leaves	Wei et al. (2021); Lin (2018); Zhang (2018); Degl et al. (2008); Vidal et al. (2019)
*Taraxacum mongolicum* Hand.-Mazz.	Asteraceae	*Taraxacum*	Temperate areas	Perennial herb	Aerial parts and roots	Nie et al. (2020); Wang et al. (2017); Chen et al. (2020)
*Sonchus brachyotus* DC.	Asteraceae	*Sonchus*	Northwest and South of China	Annual or perennial herb	Aerial parts and roots	Liu (2016)
*Sonchus oleraceus* L.	Asteraceae	*Sonchus*	Northeast, North, Central, and South China	Annual or biennial herb	Leaves	Ma et al. (2011)
*Ixeris chinensis* (Thunb.) Nakai	Asteraceae	*Ixeris*	North, South, and East China	Perennial herb	Aerial parts and roots	Dong et al. (2008)
*Hypochaeris radicata* L.	Asteraceae	*Hypochaeris*	Europe and China	Perennial herb	Flowering heads	Ye et al. (2007); Zidorn et al. (2005); Ortiz et al. (2008)
*Bidens tripartita* L.	Asteraceae	*Bidens*	China	Perennial plant	Aerial parts	Pozharitskaya et al. (2010)
*Rabdosia rubescens* (Hemsl.) Hara	Lamiaceae	*Rabdosia*	China	Small shrub	Leaves	Zhao et al. (2013)
*Orthosiphon stamineus* Benth.	Lamiaceae	*Orthosiphon*	India, Malaysia, China, Australia, and the Pacific area	Perennial herb	Leaves	Ameer et al. (2012); Guo et al. (2019b)
*Echinodorus grandiflorus*	Alismataceae	*Echinodorus*	Central America and South Brazil	Perennial marsh plant	Leaves	Han and Shi (2009); Marques et al. (2017)
*Arachis hypogaea* L.	Leguminosae	*Arachis*	Tropics and Subtropics	Annual plant	Leaf terminals	Krishna et al. (2015); Zhou and Meng (2017)
*Equisetum arvense* L.	Equisetaceae	*Equisetum*	Europe, Asia, and North America	Perennial herb	Sprouts and gametophytes	Veit et al. (1991); Veit et al. (1992); Xia (2019)
*Lygodium japonicum* (Thunb.) Sw.	Lygodiaceae	*Lygodium*	Australia and China	Perennial climbing plant	Frond	Yang et al. (2021)
*Zostera marina* L.	Potamogetonaceae	*Zostera*	Temperate northern hemisphere	Perennial herb	Leaves	Min et al. (2019); Pilavtepe et al. (2012)

#### 2.1.1 Angiospermae

Chicoric acid is widely distributed in dicotyledons of Angiospermae, namely, Asteraceae, Lamiaceae, Rosaceae, Alismataceae, Cucurbitaceae, and others. Many reports have focused on the study of *E. purpurea*, *Pterocypsela laciniata*, and *Cichorium intybus* of the Asteraceae family.

##### 2.1.1.1 Asteraceae

There are eight species and several varieties of the *Echinacea*, among which *E. purpurea*, *Echinacea angustifolia* (DC) Hell., and *Echinacea pallida* (Nutt.) are widely used in medicine. *E. purpurea* is a perennial herb with a medical history dating back more than 300 years (Shah et al., 2007) and was introduced in China as a flower in the 1970s. It is currently cultivated on a large scale in China as a medicinal plant. *E. purpurea* is widely used in drug materials, nutritional supplements, and health foods. Chicoric acid is often used as an indicator component in the quality evaluation of materials and preparations (Wills and Stuart, 1999; Xu et al., 2006; Sun, 2011; Chen et al., 2012; Han et al., 2013; Han et al., 2014; Wang et al., 2016).

There are eight species and one variety species of *Pterocypsela*, located primarily in East Asia and Southeast Asia. China is rich in resources, most of which are distributed in the east. As a common weed, *Pterocypsela* has strong fecundity and adaptability to harsh environments (He, 2012; Ke et al., 2015). *P. laciniata* is a perennial herb of *Pterocypsela* with abundant plant resources and chicoric acid.

Cichorium originated in ancient Rome and Greece and was distributed in the Mediterranean and Southwest Asia. Of the eight total species, three exist in China. *C. intybus*, a perennial herb of Cichorium, was cultivated as a high-grade vegetable in the 19th century. It can be cooked into lettuce (Carazzone et al., 2013) and used for health care. Chicoric acid is an important index for the quality evaluation of *C. intybus* (Mascherpa et al., 2012; Zhou, 2014), and the amount of chicoric acid differs significantly in different regions and in different areas of the same plant (Xu, 2008).

*Lactuca sativa*, an annual or biennial vegetable crop, is widely cultivated in temperate areas around the globe. An in-depth analysis did not reveal the origin of *L. sativa*, but it was first domesticated near the Caucasus (Wei et al., 2021). A variety of cultivation types gradually formed after long-term directional selection and cultivation (Lin, 2018), which were divided into six cultivation types (Zhang, 2018). The content of chicoric acid varied significantly in response to different storage environments (Degl et al., 2008; Oh et al., 2009; Becker et al., 2015; Vidal et al., 2019).

There are more than 2,000 species of the *Taraxacum*, widely distributed from the temperate areas in the northern hemisphere to the central subtropical regions and South America (Gong et al., 2001). Dandelion is a perennial herb of *Taraxacum*, and there are 20 species used as medicinal plants in China. Large-scale artificial cultivation has continued in China because of its low growing environment requirements, simple management techniques, and high planting yield (Chen et al., 2020). *Taraxacum mongolicum* and *Taraxacum sinicm* Kitag reportedly contain chicoric acid, but there are few studies on the topic (Wang et al., 2017; Nie et al., 2020).

There are 50 species of *Sonchus* worldwide, located mostly in Europe, Asia, Africa, and the Mediterranean/Atlantic islands (Liu, 2016). *Sonchus brachyotus*, an annual or perennial herb of *Sonchus*, sees extensive use in northwest and southern China. *S. oleraceus*, distributed in Northeast, North, Central, and Southern China (Ma et al., 2011), is not only easy to cultivate but also rich in nutrition. *Sonchus asper* (L.) Hill and *S. oleraceus* also contain chicoric acid, but the amount remains unclear.

There are 20 species of *Ixeris* in North, South, and East China. *Ixeris chinensis* and *Ixeris sonchifolia* Hance belong to *Ixeris*. The perennial herb *I. chinensis* has been used as a traditional Chinese herbal medicine for thousands of years. *I. sonchifolia* has been cultivated artificially recently in many areas. Chicoric acid is often used as one of the quality control indexes of these plants (Dong et al., 2008; Zhao and Jiang, 2016).

*Hypochaeris radicata* is a perennial herb, and studies have indicated that the oldest populations of *H. radicata* originated in Europe and expanded *via* at least three migratory routes to other countries (Ortiz et al., 2008) and have been found in Zhejiang and Guizhou of China (Ye et al., 2007). The species are largely used for both food and medicine in Italy. One study has shown a positive correlation between the altitude of the growth environment and the content of chicoric acid (Zidorn et al., 2005). *Bidens tripartita*, a perennial plant, is widely distributed throughout China. Previous studies on *B. tripartita* confirmed the presence of chicoric acid in this plant (Pozharitskaya et al., 2010).

##### 2.1.1.2 Lamiaceae

*Rabdosia rubescens*, a small shrub of *Rabdosia*, is native to the valley of the Yellow River and the Yangtze River, as well as the Jiyuan Taihang Mountain and Wangwu Mountain in Henan province. The growing environment is characterized by hillsides and woodlands. Recently, the artificial planting of *R. rubescens* in Jiyuan City has expanded, with yields accounting for 95% of the total Chinese output. *R. rubescens* in Jiyuan City is a “national geographic product.” In China, *R. rubescens* is consumed as a famous traditional medicinal herb and tea (Zhao et al., 2013). Although the plant contains chicoric acid, not much research exists on this.

*Ocimum basilicum* L., a perennial herb of the *Ocimum*, has more than 150 species around the world. *O. basilicum*, originated in the warm tropical climates of India, Africa, and southern Asia and has been cultivated worldwide as an aromatic crop and ornamental plant. Some research reported that levels of chicoric acid varied from 0.09 to 0.16 mg/g in dried samples (Kwee and Niemeyer, 2011).

*Orthosiphon stamineus* belongs to a perennial *Orthosiphon* herb, occurs widely in India, Malaysia, China, Australia, and the Pacific area. *O. stamineus* is a valued medicinal plant in traditional folk medicine (Ameer et al., 2012). Leaves of this plant find use in tea, and the rest of the dried plant is used for medicine. Chicoric acid is also the most important bioactive component of this plant (Guo et al., 2019a).

##### 2.1.1.3 Other Genera

Chicoric acid has been detected in other plants from different families, namely, *Echinodorus grandiflorus*, *Cucurbita pepo* L. (Iswaldi et al., 2013), *Arachis hypogaea*, *Pulsatilla chinensis* (Bge.) Regel (Zhang et al., 2008), and *Pyracantha fortuneana* (Maxim.) Li. However, acid levels in these plants are unknown and require additional research.

*E. grandiflorus*, a perennial marsh plant of the Alismataceae, originates from Central America and southern Brazil (Han and Shi, 2009) and has medicinal uses (Marques et al., 2017). *C. pepo* is a trailing annual herb of the Cucurbitaceae. *A. hypogaea*, an annual plant of the Leguminosae, is widely distributed in the tropics and subtropics. *A. hypogaea* plays an important role in the world agricultural economy not only for vegetable oil but also as a source of proteins, minerals, and vitamins (Krishna et al., 2015). *A. hypogaea* is the highest yielding oil crop in China (Zhou and Meng, 2017).

*P. fortuneana* is an evergreen shrub or small tree of the Rosaceae. Because of its strong adaptability, *P. fortuneana* thrives with high yields and is widely distributed in Asia and Europe. Chicoric acid was also detected in the fruit of *P. fortuneana*. *P. chinensis*, belonging to the genus *Pulsatilla* of the buttercup family, is also widely distributed in Europe and Asia. Eleven of the 43 species of this plant have been found in Liaoning, Hebei, and Henan.

#### 2.1.2 Pteridophyta

Chicoric acid may be a specific chemical component in Pteridophyta, because chicoric acid has been detected in 23 of 29 species (Hasegawa and Taneyama, 1973). Pteridaceae, Dryopteridaceae, Equisetaceae, and other Pteridophyta (Cao et al., 2013) have been cultivated for health care.

*Equisetum arvense*, a perennial herb of Equisetaceae, is native to Europe, Asia, and North America and widely distributed in Heilongjiang (Xia, 2019). Meso-chicoric acid was isolated from the sprouts (fertile) and gametophytes of *E. arvense* (Veit et al., 1991; Veit et al., 1992), but little reports about the content.

*Pteris cretica* L. and *Onychium japonicum* (Thumb.) Kze. belong to Pteridaceae. *P. cretica* is a perennial evergreen herb. *O. japonicum* occurs primarily in Taiwan, Japan, Korea, and other Asian countries and is used to treat enteritis, jaundice, flu, chronic gastritis, and fever. *Dryopteris erythrosora* (D.C. Eaton) Kuntze is a species of Dryopteridaceae native to China and Japan and distributed throughout East Asia. Chicoric acid was detected in the frond of these plants.

*Lygodium japonicum*, a perennial climbing plant of Lygodiaceae, mainly distributed in Australia and the southwestern area of China (Yang et al., 2021). The entire *L. japonicum* plant is used to treat various inflammatory diseases. *Pteridium aquilinum* (L.) Kuhn is a serious invasive weed of upland and marginal lands in many parts of the world (Stewart et al., 2008); however, chicoric acid has been detected in it.

#### 2.1.3 Other Categories

The ocean is a potential source of various raw materials for food and drugs. Compared with terrestrial plants, marine plants with large biomass have obvious advantages as a source of chemical raw materials. *Cymodocea nodosa*, *Syringodium fifiliforme* Kütz, and *Posidonia oceanica* (L.) Delile also contain chicoric acid.

*P. oceanica* occurs primarily in the Mediterranean Sea. Phenolic compounds are the major metabolites and chicoric acid accounts for 80–89% of the total phenolics. Due to the significant content of chicoric acid, more and more investigations on this plant have taken place (Haznedaroglu and Zeybek, 2007). However, this species is endangered because of anthropogenic effects. *C. nodosa* is one of the most important macrophytes in the Mediterranean Sea and eastern Atlantic coasts. Some studies have shown that the content of chicoric acid varies in different parts of the plant (Grignon-Dubois and Rezzonico, 2013).

*Zostera marina*, the most widespread seagrass species of Potamogetonaceae throughout the temperate northern hemisphere (Min et al., 2019), is the largest seagrass meadow in the Bohai Sea and Yellow Sea areas in China. Although chicoric acid has been detected in the leaves of *Z. marina*, the data are incomplete (Pilavtepe et al., 2012). *S. fifiliforme* is distributed across the Caribbean Sea and the Gulf of Mexico as seagrass, and it has been reported that *S. fifiliforme* contains chicoric acid, but no exact statistics are available (Nuissier et al., 2010).

### 2.2 Methods of Chicoric Acid Extractions and Its Contents in Plants

The amount of chicoric acid is closely related to the plant source, medicinal parts, harvest period, processing, and extraction methods. So far, a systematic study on chicoric acid levels has not been found. Although chicoric acid comes from a variety of plants, the selection of safe and economical plant sources requires further research. By analyzing the relationship between factors and the content of chicoric acid, the distribution of chicoric acid in the plant can be preliminarily predicted, which provides a basis for further development of chicoric acid.

#### 2.2.1 Content of Chicoric Acid in *Echinacea purpurea*

*E. purpurea* is the raw material for chicoric acid extraction in most studies. Several factors impact chicoric acid levels (Table 2). For 2-year old *E. purpurea*, the content of chicoric acid is higher in flowers than in other parts during flowering (Wang et al., 2002). The content of chicoric acid in the stems, leaves, and flowers were 9.7%, 44.7%, and 23.6%, respectively (Li et al., 2011). The chicoric acid levels maximized during flowering and provided the best harvest period for *E. purpurea* (Innocenti et al., 2005).

**TABLE 2:** Comparison of chicoric acid levels in *Echinacea purpurea* (L.) Moench.

Extraction methods	Medicinal origin	Medicinal parts	Extraction conditions	Yield of chicoric acid (%)	References
Reflux extraction	Shaanxi	Dried aboveground parts	Extraction was performed 3 times with 1.5 h each time	1.03	Zhong et al. (2010)
	Xinjiang	All dried grasses	15 times; 40% ethanol, 3 times, 2 h each time	0.55	Ma et al. (2014)
			The extraction temperature was 90°C		
	Guangdong	All dried grasses	8 times; 80% ethanol, 3 times, 1 h each time	1.09	Zhang et al. (2008)
	Hebei	Dried flowers	20 times; 20% ethanol, 2 times, 2 h each time, extraction temperature was 90°C	0.75	Sun et al. (2019)
	Shandong	Dried flowers	20 times; 60% ethanol, 2 times, 2 h each time, extraction temperature of 70°C	2.30	Cheng et al. (2018)
Ultrasonication extraction	Anhui	All dried grasses	8 times; 55% ethanol was extracted twice, 45 min each	1.22	Cao et al. (2010)
	Beijing	Dried roots, stems, leaves, flowers, aboveground parts	125 times; methanol–0.5% phosphoric acid (4:1) solution was extracted by ultrasound for 40 min	0.90 (root)	Wang et al. (2002)
				0.43 (stem)	
				1.84 (leaf)	
				2.15 (flower)	
				1.05 (overground part)	
	Shandong	Dried roots, stems, leaves, flowers	125 times; 70% methanol, ultrasound 30 min	1.28 (root)	Han et al. (2014)
				0.36 (stem)	
				2.32 (leaf)	
				2.11 (flower)	
	Shandong	Dried aboveground parts	62.5 times; 70% methanol was extracted by ultrasonography for 3 times, 10 min each	2.02	Xin et al. (2012)
	Guangdong	Dried roots, stems, leaves, flowers, whole grasses	62.5 times; methanol–0.5% phosphoric acid aqueous solution (4:1) was extracted by ultrasound for 60 min	1.21 (root)	Li et al. (2011)
				0.07 (stem)	
				0.56 (leaf)	
				0.25 (flower)	
				0.33 (whole herb)	
Ultrasonic microwave co-extraction method	Tianjin	Fresh roots	25 times; 50% ethanol, ultrasound for 90 s without microwave power; the extraction power was 300 W and the extraction time was 660 s	0.02	Wang et al. (2015)
Spray extraction	Shaanxi	All dried grasses	Spray 4 times 70% ethanol at 20 kg pressure for 3 min	0.53	Zhao and Yang (2018)
Supercritical carbon dioxide extraction method	Guangdong	Dried flowers	CO_2_ was extracted with 40% ethanol entrainment at a flow rate of 25 kg/hand a pressure of 30 MPa for 2 h at 60°C	1.06	Lin et al. (2011)

#### 2.2.2 Chicoric Acid Levels in Other Plant Resources

Related references have reported that the content of chicoric acid in *P. laciniata* is 26.1 mg/g, 2.2 times higher than in *E. purpurea* (Ke, 2015). Chicoric acid levels varied with different plant areas and medicinal parts in *C. intybus*, but chicoric acid levels above ground from Jiangsu were higher (Innocenti et al., 2005). The content of chicoric acid in *I. chinensis* and *S. brachyotus* varied greatly, the reason for which may be related to medicinal parts and picking time (Liu, 2016), drying process and preparation method (Sun, 2011), and extraction conditions and other factors (Xin et al., 2012). A summary of these studies appears in Table 3.

**TABLE 3:** Comparison of the content of chicoric acid in different plants.

Herbs	Medicinal origin	Medicinal parts	Extraction methods	Extraction conditions	Yield of chicoric acid (%)	References
*Cichorium intybus* L.	Xinjiang	Dried stem	Ultrasonication extraction	21 times; 50% ethanol, ultrasound at 60°C for 50 min (180 W)	0.15	Meng et al. (2015); Meng (2016)
	Jiangsu	Dried overground part	Ultrasonication extraction	12 times; 54% ethanol, ultrasound 30 min (40 w)	0.15	Xu (2008)
	Jiangsu	Dried overground part	Reflux extraction	24 times; 54% ethanol was refluxed at 90°C for 1 h	0.15	Xu (2008)
	Netherlands	Dried overground part	Dried overground part	Reflux of 60 times 70% methanol at 60°C for 1 h	0.88	Hua et al. (2011)
*Ixeris chinensis* (Thunb.) Nakai	Shanxi	Dried overground part	Reflux extraction	50 times; 70% ethanol heated reflux extraction 1 h	0.77–1.14	Liu (2016)
*Sonchus brachyotus* DC.	Shanxi	Dried overground part	Reflux extraction	50 times; 70% ethanol heated reflux extraction 1 h	0.34–2.69	Liu (2016)
*Pterocypsela laciniata* (Houtt.) Shih	Jiangsu	Dried leaves	Ultrasonication extraction	Ultrasonic extraction with 100 times 80% ethanol solution at 45°C for 70 min	2.61	Liu (2016)
*Sonchus asper* (L.) Hill	Jiangsu	Dried leaves	Ultrasonication extraction	Ultrasonic extraction with 100 times 80% ethanol solution at 45°C for 70 min	1.50	Ke (2015)

## 3 Chemical Synthesis of Chicoric Acid

*E. purpurea* is often used as a crude material for chicoric acid production, but a large-scale preparation of high purity chicoric acid has not been reported, which limits its further development and utilization. This requires a synthetic method as an alternative to complement natural plant extraction. In this article, synthetic methods to produce chicoric acid are summarized and the characteristics of each method are compared to provide new synthetic routes.

### 3.1 Chicoric Acid Synthesis Using (E)-3-(2-Oxo-3a,7a-Dihydrobenzo[d][1,3]Dioxol-5-yl)Acryloyl Chloride and (2R,3S)-2,3-Dihydroxysuccinic Acid

**FIGURE 3:**
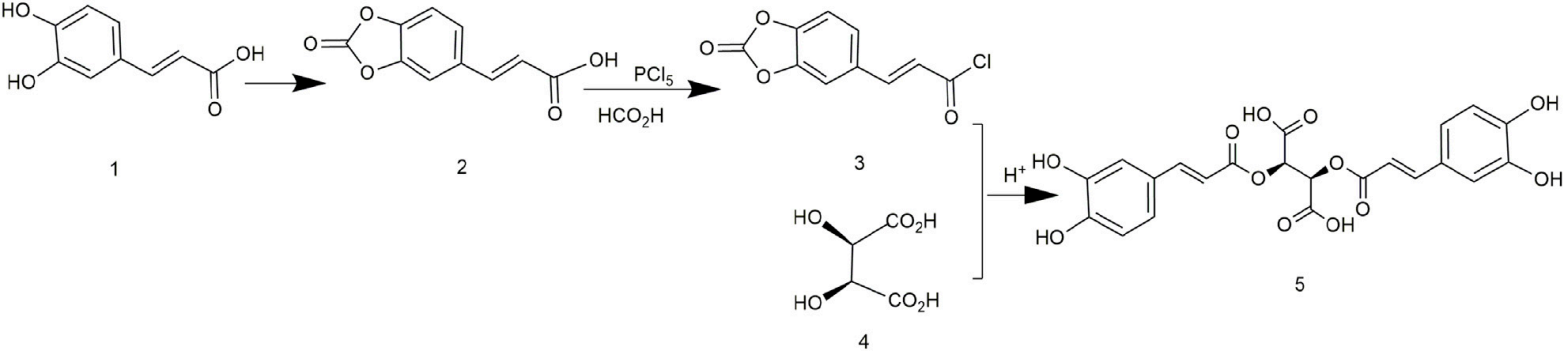
Chemical synthesis pathway 1 of chicoric acid.

In 1958, chicoric acid was first extracted from *C. intybus* and its different configurations were chemically synthesized. L-chicoric acid was synthesized *via* caffeoyl chloride and D-tartaric acid and deprotection of acetic acid (Figure 3). D-chicoric acid, L-chicoric acid, and meso-chicoric acid were synthesized by reactions with L-tartaric acid, D-tartaric acid, and meso-tartaric acid, respectively. These syntheses were simple, but the purity of chicoric acid was too low. In addition, the use of the unstable caffeoyl chloride led to low yields of chicoric acid (Scarpati and Oriente, 1958).

### 3.2 Chicoric Acid Synthesis Using Di-Tert-Butyl (2R,3S)-2,3-Dihydroxysuccinate and (E)-4-(3-Chloro-3-Oxoprop-1-En-1-yl)-1,2-Phenylene Diacetate

**FIGURE 4:**
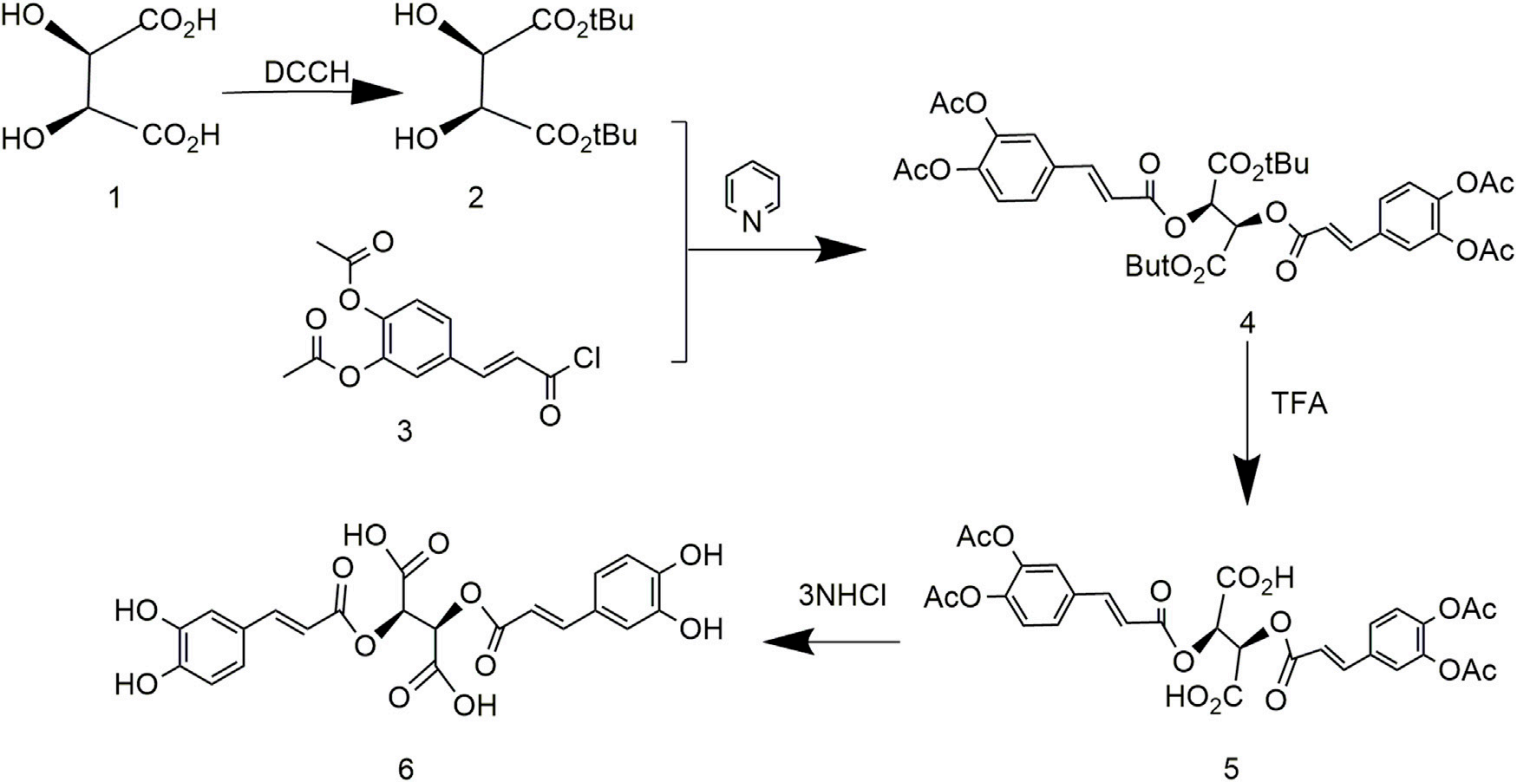
Chemical synthesis pathway 2 of chicoric acid.

Based on previous research, the optimized process simplified production and occurred under mild reaction conditions (Kulangiappar et al., 2014). First, tert-butyl tartrate was obtained by protecting the carboxyl group of tartaric acid with a tert-butyl group followed by protecting the phenolic hydroxyl of caffeic acid with an acetyl group to produce diacetyl caffeic acid. Then, compound 2 reacted with compound 3, and finally the protective group of previous reaction was removed to obtain L-chicoric acid (Figure 4). The reaction conditions of this method are mild, but L-tert-butyl tartrate is difficult to synthesize. Many by-products and low yields limit large-scale production.

### 3.3 Chicoric Acid Synthesis Using Dibenzhydryl (2R,3R)-2,3-Dihydroxysuccinate and (Z)-4-(3-Chloro-3-Oxoprop-1-En-1-yl)-1,2-Phenylene Dimethyl Bis(Carbonate)

**FIGURE 5:**
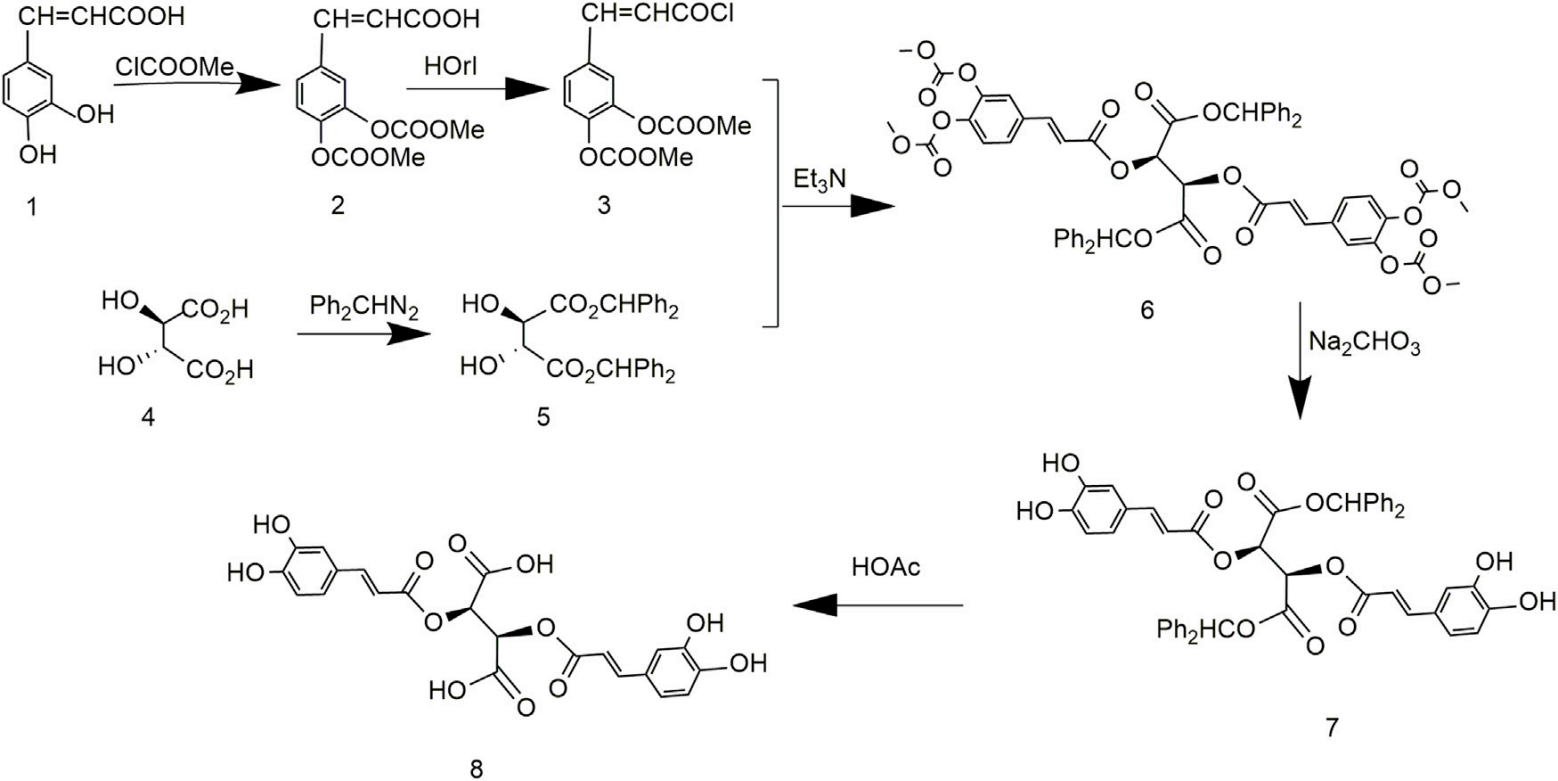
Chemical synthesis pathway 3 of chicoric acid.

King et al. (1999) reported a method for synthesizing chicoric acid with different configurations by protecting tartaric acid with diphenylmethyl. The synthesis of chicoric acid was mainly carried out in two directions (Figure 5). Diphenylmethane protected the carboxyl group of L-tartaric acid, and the phenolic hydroxyl group of caffeic acid was protected by ClCOOMe to obtain 3,4-cyclocarbonate of caffeoyl chloride. The two protected components reacted, and the protecting groups were removed to obtain L-chicoric acid. Too many steps lead to an overall yield of 33.3%. In addition, the carboxyl-protected crude material of tartaric acid diphenyl diazomethane was unstable to the point of explosion during synthesis, which seriously curtails industrial adaptation.

### 3.4 Chicoric Acid Synthesis Using Dibenzyl (2R,3R)-2,3-Dihydroxysuccinate and (E)-3-(3,4-Bis(Benzyloxy)Phenyl)Acrylic Acid

**FIGURE 6:**
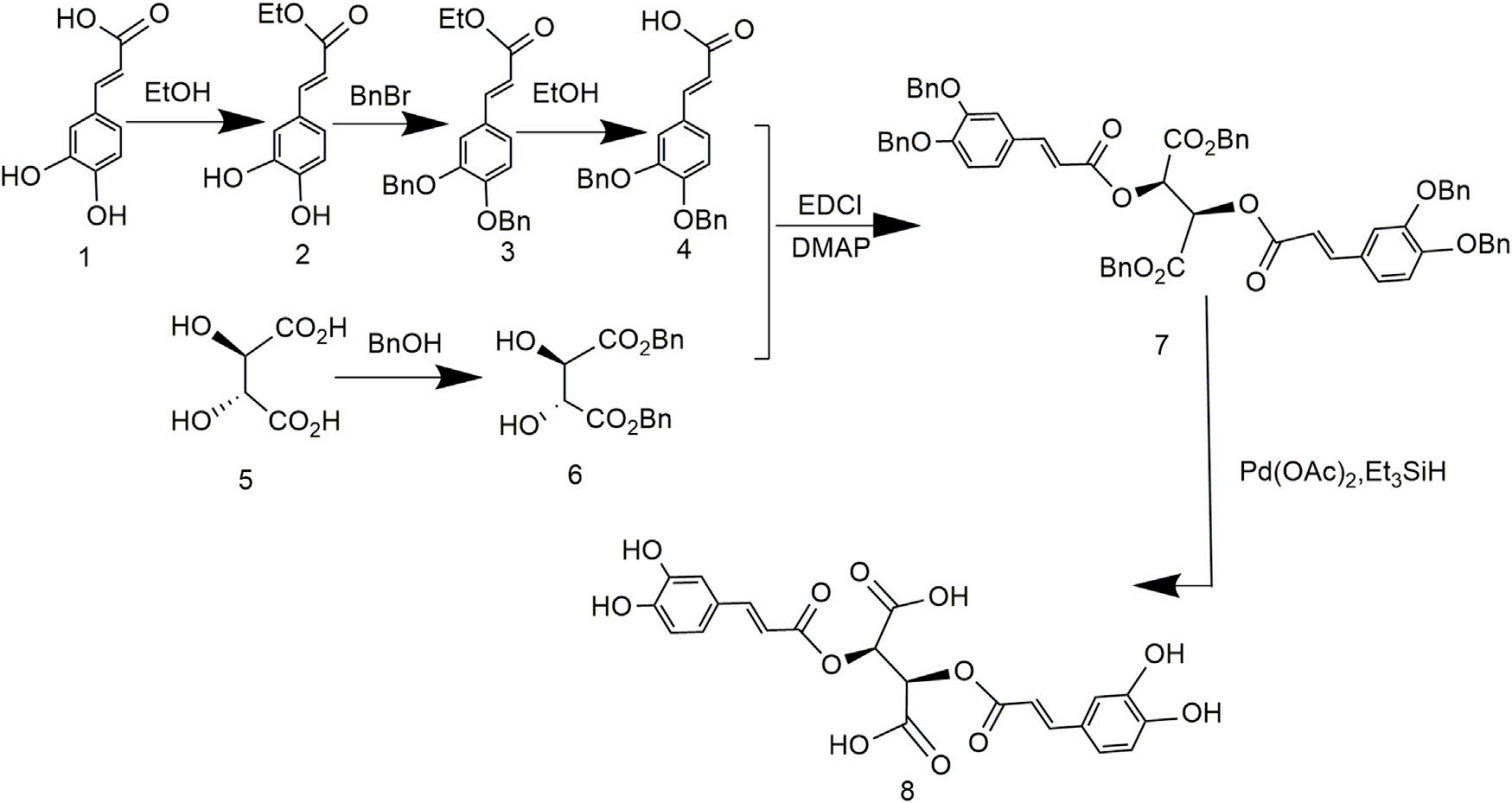
Chemical synthesis pathway 4 of chicoric acid.

To address problems caused by expensive tartaric acid derivatives and poor reproducibility, the synthesis of chicoric acid was further optimized. Both carboxyl groups of tartaric acid and the phenolic hydroxyl group of caffeic acid were protected by benzylation, followed by esterification and reduction to obtain L-chicoric acid (Figure 6). This synthetic route used heavy metal salts, which increased the production cost and led to heavy metal residues. In addition, the by-products affected drug quality (Lamidey et al., 2002).

### 3.5 Chicoric Acid Synthesis Using (E)-3-(3,4-Diacetoxyphenyl)Acrylic Acid and (2R,3R)-2,3-Dihydroxysuccinic Acid

**FIGURE 7:**
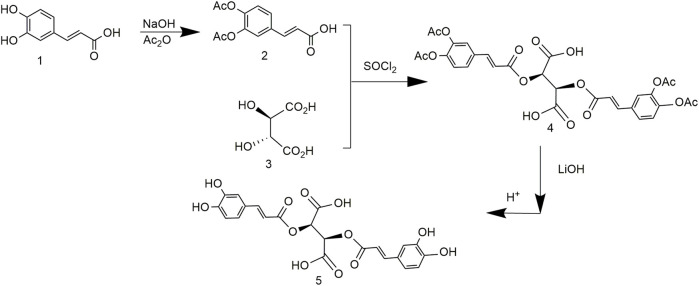
Chemical synthesis pathway 5 of chicoric acid.

Chicoric acid synthesis was improved based on a previous work to simplify the route and improve the purity (Zhou, 2014). The phenolic hydroxyl of caffeic acid was protected by an acetyl group and reacted with L-tartaric acid to obtain (2R,3R)-2,3-bis(((E)-3-(3,4-diacetoxyphenyl)acryloyl)oxy)succinic acid. LiOH hydrolysis removed the protecting group; the metal ions and chicoric acid formed a stable complex and were hydrolyzed to obtain relatively pure chicoric acid (Figure 7). Although the purity of the product was 99.7%, it did not maximize the yield or reduce the cost.

### 3.6 Chicoric Acid Synthesis Using (E)-Hypochlorous (E)-3-(2-Oxidobenzo[d][1,3,2]Aioxathiol-5-yl)Acrylic Anhydride and (2R,3R)-2,3-Dihydroxysuccinic Acid

**FIGURE 8:**
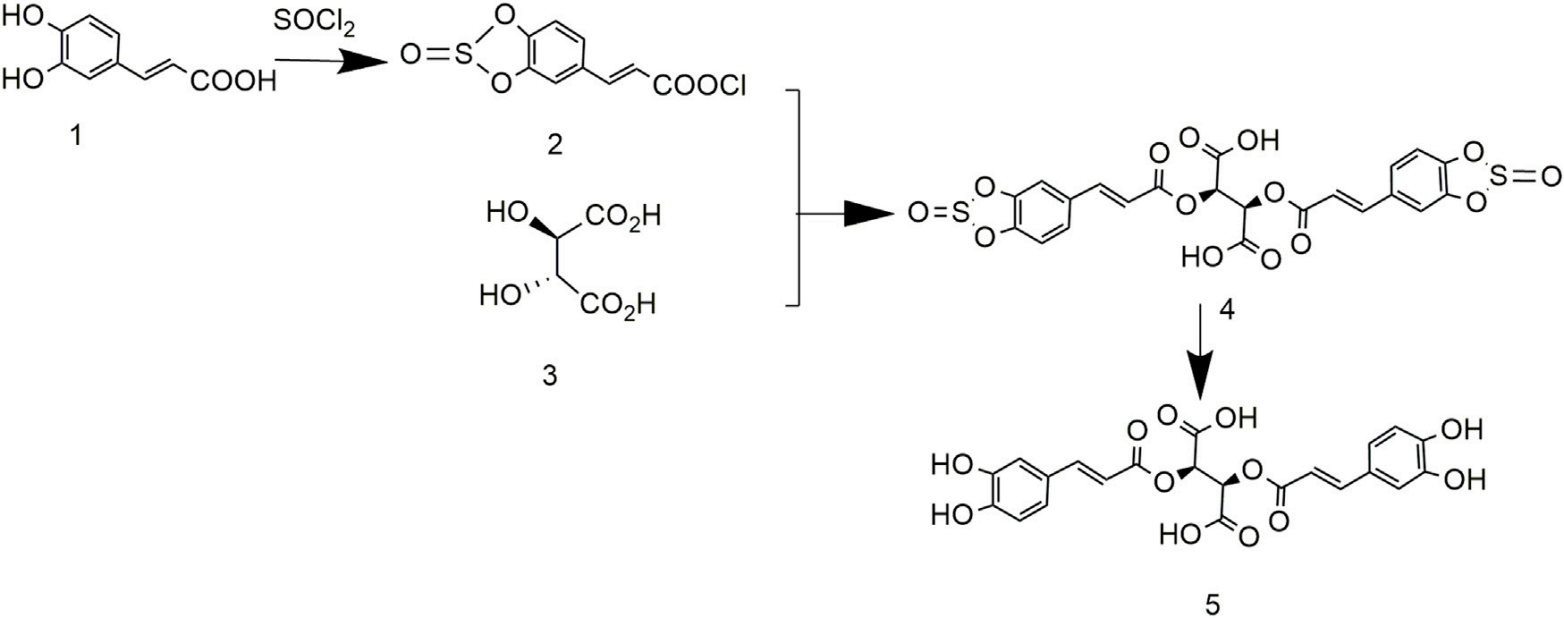
Chemical synthesis pathway 6 of chicoric acid.

To solve the problem of high cost and low yield, the synthesis of chicoric acid was optimized, and its crystal shape studied (Tian et al., 2021). The product of caffeic acid and sulfoxide chloride reacted with L-tartaric acid to yield L-gesnerate sulfonate. After removing the protecting group in an alkaline solution, L-chicoric acid was obtained (Hunan Normal University, Changsha, 2021; Figure 8). After further crystallization, the yield and purity of chicoric acid were 80.5% and 98.5%, respectively. Although the purity and yield of the product increased, the process was still cumbersome.

The structure of chicoric acid was obtained by intermolecular esterification of tartaric acid and two molecules of caffeic acid. Because both tartaric acid and caffeic acid contained hydroxyl and carboxyl groups, side reactions readily occurred. Therefore, the most common synthetic pathway utilized protective groups to protect phenolic hydroxyl groups of caffeic acid and carboxyl groups of tartaric acid before esterification. The difference lies in the selection of raw materials and the different reagents to protect phenolic hydroxyl groups of tartaric acid. These protecting groups include tert-butyl, benzyl, or carboxyl benzyl to protect tartaric acid, while the caffeic acid phenol hydroxyl was protected by acetyl, benzyl ethyl, oxygen acyl, or metal ions. Despite the myriad synthetic methods, none brought higher quality chicoric acid in high purity, yield, and economic and environmental protection.

## 4 Biosynthesis of Chicoric Acid

Chicoric acid is a phenolic acid and generally forms via the shikimic acid‐phenylpropanoid pathway (Kuhnl et al., 1987; Petersen et al., 2009). Understanding what factors regulate the distribution of chicoric acid in plants may help regulate chicoric acid accumulation by altering certain conditions. Although there are many studies on the biosynthetic pathway of chicoric acid, its mechanism remains unclear.

**FIGURE 9:**
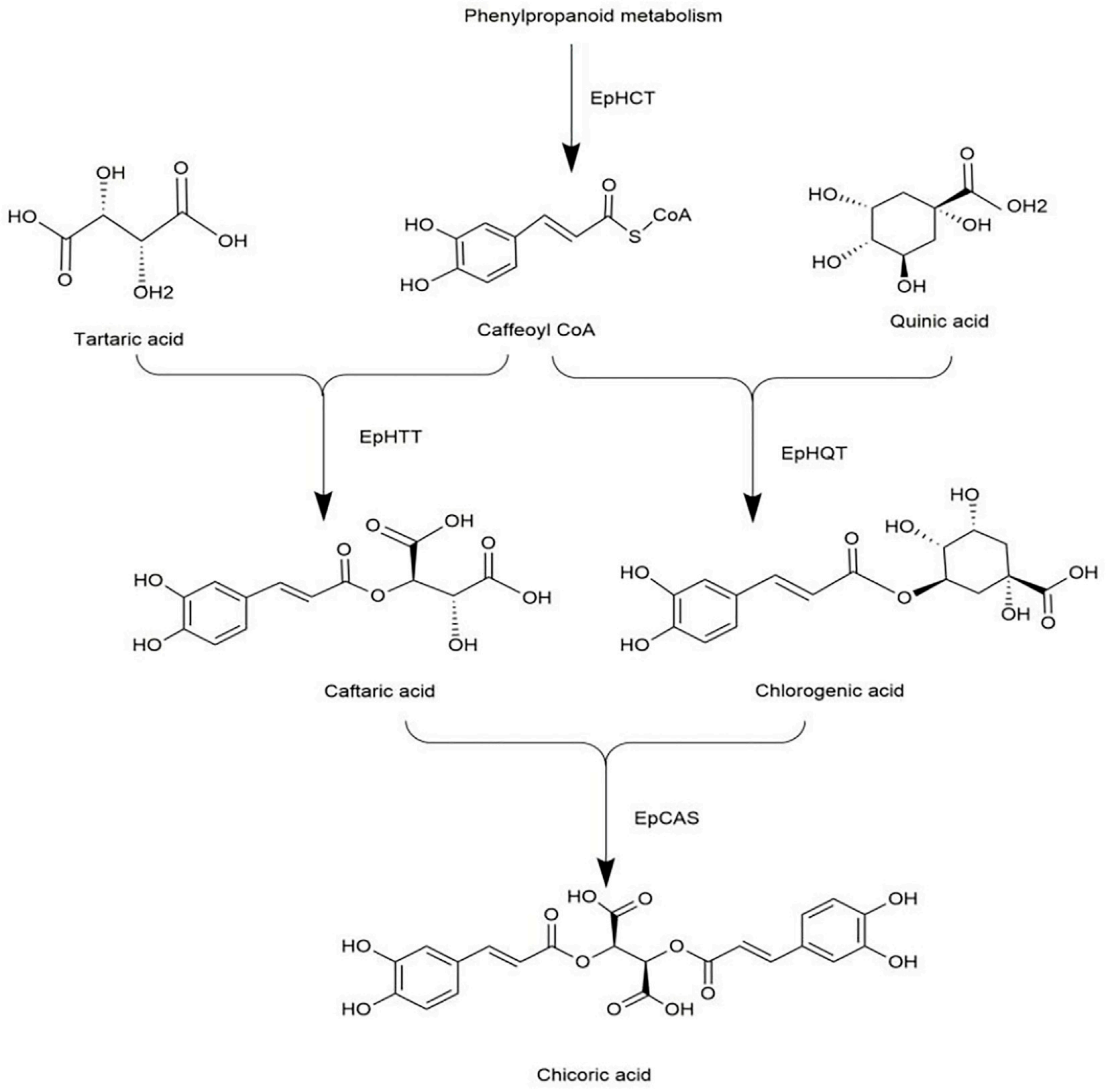
Biosynthetic pathway of chicoric acid.

Recently, a study filled this gap by reporting the biosynthetic pathway of chicoric acid in *Echinacea* (Fu et al., 2021). The biosynthesis of chicoric acid occurs in three main stages. Firstly, phenylpropanoid reacts to form caffeoyl CoA *via* the enzyme EpHCT. Secondly, EpHTT and EpHQT were responsible for the biosynthesis of caftaric acid and chlorogenic acid in the cytosol, respectively. Finally, caftaric acid and chlorogenic acid were transferred from the cytosol into vacuole, and chicoric acid was synthesized *via* EpCAS (Figure 9). The biosynthetic pathway of chicoric acid production has been reprogrammed in tobacco, but its applicability in other species needs additional study.

## 5 Bioactive Effects

Chicoric acid has a long history of clinical use and can effectively treat a variety of diseases. Because of this, numerous studies that have focused on chicoric acid have been conducted on the biological activities of both *in vitro* and *in vivo* models. Chicoric acid has long attracted attention as a medication and nutraceutical to improve health based on its anti-inflammation (Liu, 2016; Liu et al., 2017a; Liu et al., 2017b; Tsai et al., 2017; Liu et al., 2019; Li et al., 2020), glucose and lipid homeostasis (Kim et al., 2017; Lipchock et al., 2017), neuroprotection effects (Bekinschtein et al., 2008; Kour and Bani, 2011a), anti-aging effects (De Winter, 2015; Peng et al., 2019), and antioxidant and immune-stimulating properties (Kour and Bani, 2011b; Schlernitzauer et al., 2013; Chen et al., 2017; Wang et al., 2017; Jia et al., 2018; Ma et al., 2018). In addition, its antivirus properties, such as immunodeficiency viruses (Healy et al., 2009; Crosby et al., 2010; Nobela et al., 2018), herpes simplex viruses (Langland et al., 2018), and respiratory syncytial virus (Zhang et al., 2021) are particularly significant. Tables 4 and 5 summarize these studies.

**TABLE 4:** *In vitro* effects of chicoric acid in the treatment of various disorders.

Disorders	Models	Dose (μM)	Duration (h)	Effects	Suggested mechanisms	References
Diabetes	HUVECs	100	24	↓ cell apoptosis	(+) the AMPK signaling pathway; ↓ Iκ-Bα; ↓ NF-κB; ↓ iNOS; ↓ IL-1β; ↓ p-eNOS	Ma et al. (2021)
				↓ p65 NF-κB nuclear translocation		
				↓ oxidative/nitrative stresses		
	PC-12 cells	10 and 20	24	↓ misfolding; ↓ fibrillation of hIAPP; ↓ aggregation	↓ Cytotoxicity; ↑ biocompatibility	Luo et al. (2020)
Lipid metabolism	HepG2 human hepatoma	100 and 200	24	↓ lipid accumulation	(−) SREBP-1/FAS signaling pathways; (+) PPARa/UCP2 signaling pathways	Mohammadi et al. (2020)
	HepG2 human hepatoma	10 and 20	24	↓ lipid accumulation; ↓ oxidative stress; ↓ inflammation	↑ AMPK; ↑ Nrf2; ↓ NF-κB	Ding et al. (2020)
Inflammation	PBMCs (T2DM patients)	50	6	↓ inflammation	↓ IL-6; ↑ SIRT1; ↑ pAMPK	Sadeghabadi et al. (2019)
	SH-SY5Y cells	80	12	(−) inflammatory factor release; ↑ mitochondrial function and energy metabolism	↑ PGC-1α; ↑ SIRT1; ↑ pAMPK	Liu et al. (2019)
Gastric function	Human gastric cancer cell	20	48	(−) apoptosis in gastric cancer cells; ↓ cell viability	↑ p70S6 kinase; ↑ AMPK; ↑ PERK; ↑ ATF4	Sun X S et al. (2019)

↑, increase; ↓, decrease; (+), active; (−), inhibit; N/A, not available; HUVECs, human umbilical vein endothelial cells; NF-κB, nuclear factor-kappa B; Iκ-Bα, inhibitor kappa B alpha; iNOS, inducible nitric oxide synthase; IL-1β, interleukin-1 beta; hIAPP, human islet amyloid polypeptide; AMPK, AMP-activated protein kinase; Nrf2, nuclear factor–erythroid 2 related factor 2; PBMCs, peripheral blood mononuclear cells; T2DM, type 2 diabetes mellitus; IL6, interleukin 6; PGC-1α, peroxisome proliferator–activated receptor-γ coactivator-1α; SIRT1, silent information regulator type 1; pAMPK, phospho-AMP–activated protein kinase; PERK, protein kinase RNA-like ER kinase; ATF4, activating transcription factors 4.

**TABLE 5:** *In vivo e*ffects of chicoric acid in the treatment of various disorders.

Disorders	Species (sex)	Models	Dose (mg/kg/d)	Duration (days)	Effects	Suggested mechanisms	References
Brain function	C57BL/6 mice (M)	Parkinson's disease (MPTP)	40, p.o.	12	↑ immunological response	↑ BDNF; ↑ DA; ↑ 5-HT	Wang et al. (2022)
	Chicken embryo (N/A)	Neurotoxicity (TH)	100 µg/60 g (air cell injection)	19	↑ Antioxidant; ↑ anti-inflammatory; ↑ genoprotective; ↑ antiapoptotic; ↓ NO; ↓ MPO	↓ TNF-α; ↓ IL-1β; ↓ CASP3; ↓ BCL-2; ↓ NF-κB1	Farag et al. (2021)
Liver function	C57BL/6 mice (M)	Acute liver injury (LPS + d-GalN)	50, p.o.	1	↓ Hepatic injury; ↓ inflammation	(+) Nrf2 pathway; ↓ MAPKs; ↓ NF-κB; ↓ ALT; ↓ AST; ↑ AMPK	Li et al. (2020)
	Wistar rats (M)	Liver injury (methotrexate)	25 and 50, p.o.	19	↓ Hepatic injury; ↓ inflammation; ↓ oxidative stress	(+) Nrf2/HO-1 signaling and PPARγ; ↑ Nrf2; ↑ HO-1; ↑ NQO-1; ↑ PPARγ; ↑ BCL-2; ↓ Bax; ↓ cytochrome c; ↓ caspase-3	Hussein et al. (2020)
	C57BL/6 mice (M)	Nonalcoholic fatty liver (high-fat diet)	15 or 30, p.o.	63	↓ lipid accumulation; ↓ oxidative stress; ↓ inflammation	↑ SOD; ↓ ROS; ↑ AMPK; ↑ Nrf2; ↓ NF-κB	Ding et al. (2020)
Aging	*Caenorhabditis elegans* (N/A)	Lifespan extension (chicoric acid)	25 and 50, p.o.	12	↑ Oxidative stress resistance; ↓ ROS; ↓ pumping rate; ↓locomotive activity	In part through regulation AAK-2 and SKN-1	Peng et al. (2019)
Kidney function	Wistar rats (M)	Acute kidney injury (methotrexate)	25 and 50, i.p.	15	(−) apoptosis; ↑ antioxidant defenses	↓ NF-κB; ↓ p65; ↓ NLRP3; ↓ caspase-1; ↓ IL-1β; ↓ caspase-3; ↑ BCL-2	Abd EI-Twab et al. (2019)
Lung function	BALB/c mice (M)	Acute lung injury (lipopolysaccharide)	20 or 40, i.p.	12	↓ protein leakage; ↓ lung wet/dry ratio; ↑ antioxidant defenses	↓ MAPK; ↑ SOD; ↑ HO-1; ↑ Nrf2;↓ MPO	Ding et al. (2019)

M, male; F, female; ↑, increase; ↓, decrease; (+), active; (−), inhibit; p.o., *per os* (oral administration); i.p., intraperitoneal injection; N/A, not available; BDNF, brain-derived neurotrophic factor; MPTP, 1-methyl-4-phenyl-1,2,3,6-tetrahydropyridine; DA, dopamine; 5-HT, 5-hydroxyindoleacetic acid; TH, thiacloprid; TNF-α, tumor necrosis factor-alpha; NO, nitric oxide; MPO, myeloperoxidase; CASP3, apoptosis-related cysteine peptidase; BCL-2, B-cell CLL/lymphoma 2; LPS, lipopolysaccharide; d-GalN, d-galactosamine; MAPKs, mitogen-activated protein kinases; AST, aspartate aminotransferase; ALT, alanine aminotransferase; AMPK, AMP-activated protein kinase; HO-1, heme oxygenase-1; PPARγ, proliferator-activated receptor gamma; SOD, serum superoxide dismutase; ROS, reactive oxygen species; ROS, reactive oxygen species; AAK-2, a homolog of adenosine monophosphate (AMP)–activated protein kinase; SKN-1, a homolog of nuclear factor–erythroid 2 related factor 2; MPO, inflammatory cell infiltration, myeloperoxidase.

## 6 Conclusion and Perspectives

Although there are many chemical synthetic methods, the environmentally friendly and economic synthesis method for bulk preparation of chicoric acid with high purity and high yield needs optimization. Chicoric acid is unstable and the solubility of chicoric acid varies greatly in different solvents. Therefore, the stability and solubility of chicoric acid can be improved by a structural modification to preserve the activity of chicoric acid to meet the needs of different dosages. The biosynthesis of chicoric acid in *E. purpurea* is still at the preliminary research stage, and the mechanism also requires additional study to clarify. The enrichment of chicoric acid in different plants, parts, and stages can be further explained *via* an in-depth study of its biosynthesis to regulate and improve the content of chicoric acid by modern biotechnology. Even though there are an increasing number of studies reporting bioactivities of chicoric acid, there are still limitations in arriving at a concrete conclusion due to differences in models, doses, and treatment durations used. Thus, research on chicoric acid requires additional study, and in-depth studies on the pharmacodynamic mechanism are needed to guide clinical medication.

L-chicoric acid was isolated from *E. purpurea* and *P. chinensis* (Zhang et al., 2008), D-chicoric acid from *C. intybus*, and meso-chicoric acid from *E. arvense* (Veit et al., 1991; Veit et al., 1992), but the optical isomers of chicoric acid from different plants have not been systematically summarized and their pharmacological activities of different optical isomers need to be further studied. *C. intybus*, *L. sativa*, *E. purpurea*, and other herbs belong to medicinal and food homologous plants. The amount of chicoric acid in *P. laciniat*a was significantly higher than in *E. purpurea*. Some marine resources with large biomasses require more study and utilization. New medicinal resources of chicoric acid should be expanded using resource survey, biological relationships, pharmacological activity, and similarity of the growing environment. It is necessary to systematically study the related factors affecting chicoric acid levels, look for dominant species, and formulate a good scientific agriculture practice based on the biosynthesis and accumulation mechanism of chicoric acid. At the same time, a combination of modern technology and new methods such as tissue culture and biotechnology should help optimize the synthesis of chicoric acid.
