# Supplementary material for: Chicoric Acid: Natural Occurrence, Chemical Synthesis, Biosynthesis, and Their Bioactive Effects
Source: Front Chem. 2022 Jun 23;10:888673. doi: 10.3389/fchem.2022.888673 (PMC9262330; doi:10.3389/fchem.2022.888673)
Supplement: Supplementary file 1 [file DataSheet1.PDF]

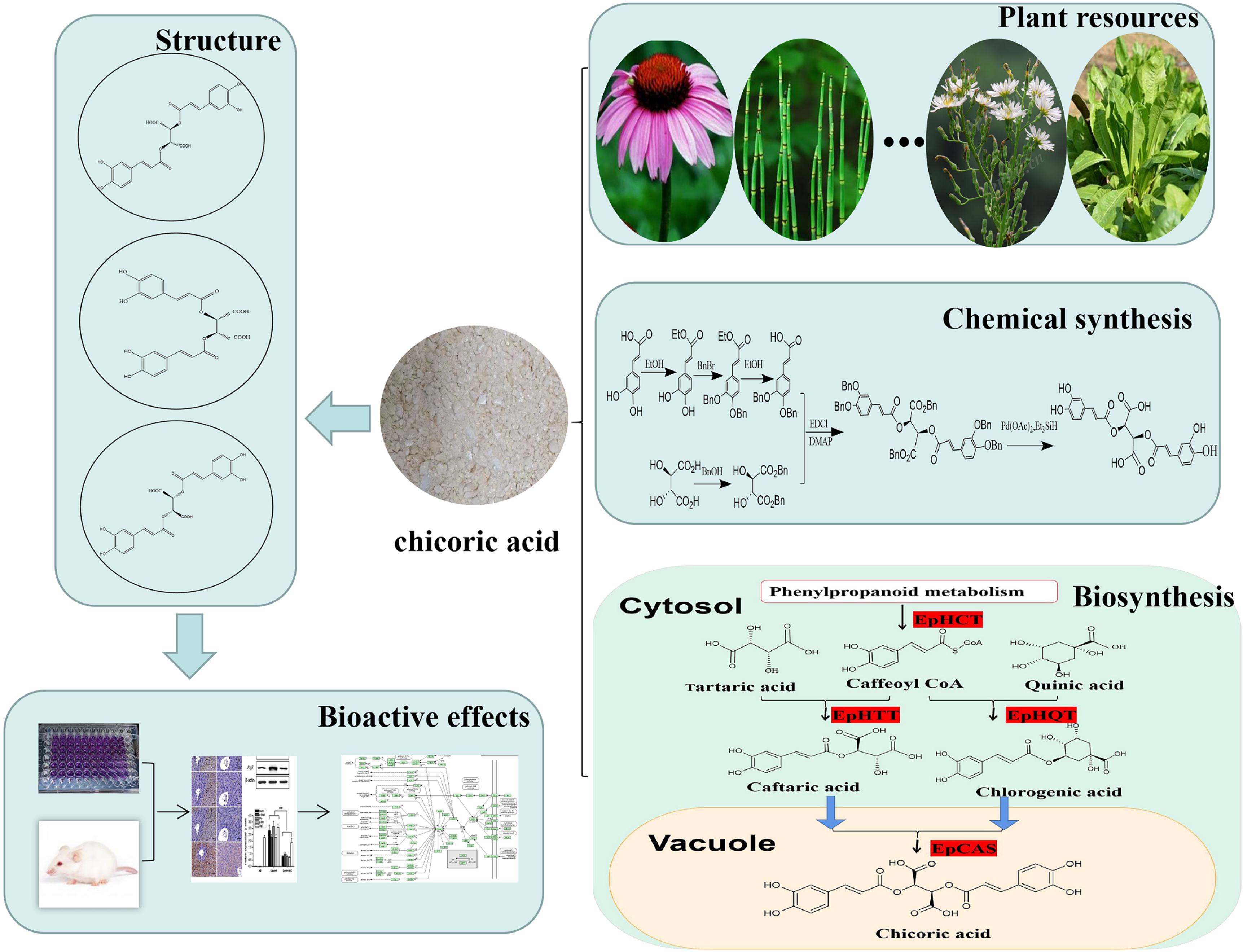
Structure
HOOC
COOH
HO
OH
chicoric acid
Plant resources
Chemical synthesis
EtOH
BnBr
EtOH
EDCI
DMAP
$Pd(OAc)_2$,Et_3SiH
BnOH
Biosynthesis
Cytosol
Phenylpropanoid metabolism
EpHCT
Tartaric acid
Caffeoyl CoA
Quinic acid
EpHTT
EpHQT
Caftaric acid
Chlorogenic acid
Vacuole
EpCAS
Chicoric acid
Bioactive effects
